# A65 VISCERAL FAT INDICES: DO THEY HELP DIFFERENTIATE CROHN’S DISEASE AND INTESTINAL TUBERCULOSIS IN CHILDREN?

**DOI:** 10.1093/jcag/gwac036.065

**Published:** 2023-03-07

**Authors:** A Srivastava, J Seetharaman, R R Yadav, S Singh, P Mishra, M S Sarma, U Poddar

**Affiliations:** 1 Paediatric Gastroenterology; 2 Department of Radiodiagnosis; 3 Department of Biostatistics , Sanjay gandhi Postgraduate Institute, Lucknow, India

## Abstract

**Background:**

Crohn’s disease (CD) and Intestinal tuberculosis (ITB) mimic each other and are often difficult to differentiate. A correct and prompt diagnosis is essential for a good outcome. Mesenteric fat hypertrophy is a feature of CD and studies in adults have shown higher visceral fat in CD than ITB. There is no published data in children.

**Purpose:**

This study evaluated the utility of visceral fat indices in differentiating CD and ITB in children.

**Method:**

Symptomatic children diagnosed to have CD or ITB based on standard recommended criteria were enrolled. The clinical and laboratory details were noted. Abdominal fat was measured on Computed Tomography in supine position at the level of L4 vertebrae. Visceral fat (VF) and subcutaneous fat (SF) area was measured separately by an experienced radiologist, blinded to the diagnosis. Sum of VF and SF was taken as total fat (TF). VF/SF and VF/TF ratio was calculated.

**Result(s):**

34 children [14 boys, median age 14.0 (inter quartile range 10.8-17.0)] years were recruited, of which 12 (29%) had CD [7 boys, age 13.0 (IQR 9.25-16.5) years] and 22 (71%) had ITB [7 boys, age-14.5 (IQR 11-17) years]. Visceral fat area (VF) was significantly higher in CD compared to ITB (table 1). However, there was no significant difference in the SF (2199.5 (1537.6-3881.6) vs 2176.5 (671.0-6651.5) mm^2^; p=0.958) and TF (3096.1 (2108.2-5373.5) vs 4518.2 (2677.6-8456.3) mm^2^; p=0.245) in ITB and CD respectively. The ratio of VF/SF and VF/TF was significantly higher in CD as compared to ITB for all cases (table1). When comparing CD and ITB in boys and girls separately the same trend was observed but the difference was statistically significant only for boys. On ROC analysis, VF:SF ratio of 0.609 predicted CD with the sensitivity of 75% and specificity of 86.4% (area under curve [AUC]-0.795, 95% CI 0.636-0.955; p=0.005). VF:TF ratio of 0.379 had similar sensitivity of 75% and specificity of 86.4% (AUC-0.795, 95% CI 0.636-0.955; p=0.005). The VF area of 1485.26 mm^2^ had a sensitivity of 83.3% and specificity of 72.7% for CD (AUC-0.758, 95% CI 0.590-0.925; p=0.01).

**Image:**

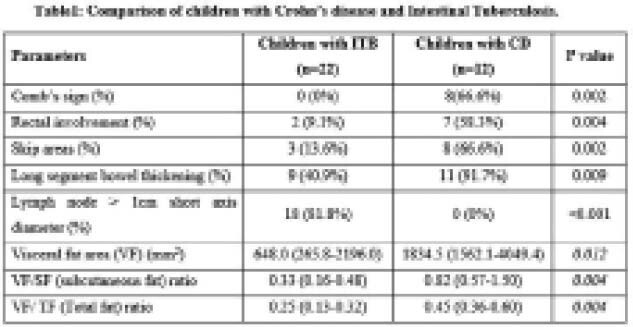

**Conclusion(s):**

The VF/SF ratio is a simple, non-invasive, objective parameter to differentiate CD and ITB in children with a good sensitivity and specificity.

**Please acknowledge all funding agencies by checking the applicable boxes below:**

None

**Disclosure of Interest:**

None Declared

